# A Unique Case of Malignant Pleuropericardial Effusion: HHV-8-Unrelated PEL-Like Lymphoma—A Case Report and Review of the Literature

**DOI:** 10.1155/2014/436821

**Published:** 2014-03-04

**Authors:** Farhan Mohammad, Muhammad Neaman Siddique, Faraz Siddiqui, M. Popalzai, Masoud Asgari, Marcel Odaimi

**Affiliations:** ^1^Department of Internal Medicine, Staten Island University Hospital, Staten Island, NY 10305, USA; ^2^Department of Hematology-Oncology, Staten Island University Hospital, Staten Island, NY 10305, USA; ^3^Department of Pathology and Laboratory Medicine, Staten Island University Hospital, Staten Island, NY 10305, USA

## Abstract

Primary effusion lymphoma (PEL) or body cavity lymphoma is a rare type of extra nodal lymphoma of B-cell origin that presents as lymphomatous effusion(s) without any nodal enlargement or tumor masses. It belongs to the group of AIDS related non-Hodgkin's lymphomas. First described in 1996 in HIV infected individuals who were coinfected with Kaposi's sarcoma-associated herpesvirus (KSHV) or HHV-8 virus, it was included as a separate entity in WHO classification of tumors of hematopoietic and lymphoid tissue in the year 2001. The definition included association with HHV-8 virus as a mandatory diagnostic criterion. However, cases were later reported where PEL-like disease process was diagnosed in HHV-8 negative patients. This was eventually recognized as a rare but distinct entity termed as “HHV-8-unrelated PEL-like lymphoma”. Herein, we are reporting a case of an elderly patient who presented with a large pleuropericardial effusion and was eventually diagnosed with this entity. Till date, only around 50 cases of HHV-8-unrelated PEL-like lymphoma have been reported and our case being EBV, HIV, and Hepatitis C negative makes it very unique and rare occurrence. We are also presenting a review of relevant literature focused mainly on comparing outcomes in patients treated with and without chemotherapy.

## 1. Case Presentation

A 76-year-old ex-smoker male with past medical history of hypertension and atrial fibrillation presented with exertional dyspnea and a recent weight loss of 15 lbs. He denied substance abuse. Review of systems and EKG were negative. On physical examination, breath sounds at bilateral lung bases were decreased. The superficial lymph nodes, liver, and spleen were not palpable on physical examination. There was no lower extremity edema. Hemogram revealed a WBC count of 10,700 cells/mm^3^ with 80% granulocytes, hemoglobin of 12.3 g/dL, and platelet count of 303000/microl. Cardiac enzymes were normal. Thyroid function tests were normal. Chemistry showed mild elevation of alkaline phosphatase and GGT. Lactate dehydrogenase was 158 U/L and ESR was 23 mm/hr. Chest radiography showed enlarged cardiac silhouette and small bilateral pleural effusions. Echocardiography showed a large pericardial effusion. He underwent pericardiocentesis with removal of 800 mL of hemorrhagic fluid. Symptoms improved and patient was discharged home to follow up as an outpatient. Pericardial fluid analysis revealed 12,300 leukocytes with 90% monocytes, 246,000 erythrocytes, a protein of 4.7 g/dL, and LDH of 6,000 IU/L. The fluid was cellular and cytology showed atypical lymphocytes. Many cells were strongly positive for CD 45 and B-cell antigens, CD79a, CD 20, and PAX 5. There was no significant expression of CD10, CD30, CD138, bcl-1, bcl-6, MUM-1, calretinin, or Ber-EP4. There was no significant expression for Epstein-Barr virus (EBV). Later, he developed a purpuric rash over the extremities. Viral serologies for HIV, HCV, HHV-8, Lyme disease, and EBV were negative. CRP was 4.8 mg/dL, but workup for SLE, rheumatoid arthritis, Sjogren's syndrome, MCTD, Wegener's granulomatosis, antiphospholipid syndrome, and paraproteinemia was negative.

**Figure 1:**
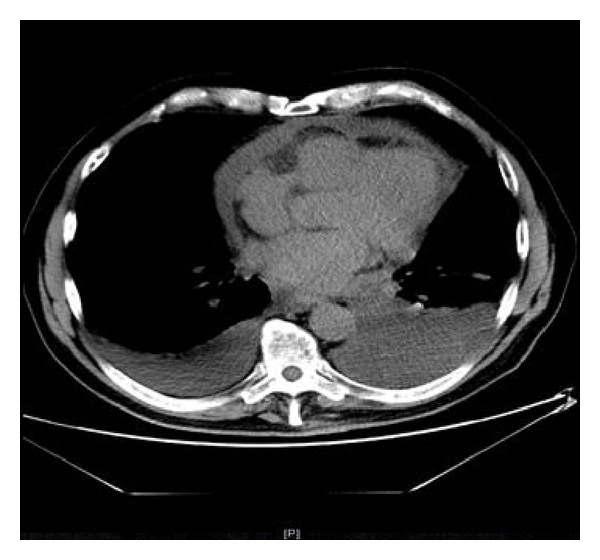
CT scan of chest with Pleuropericardial effusion. CT scan showing large Pleuropericardial effusion.

A month later CT chest was done as outpatient for recurrence of symptoms.

(Figure 1) It showed recurrent small pericardial effusion and a large left-sided pleural effusion. Pleural fluid was drained. It was hemorrhagic and analysis revealed 6800/mm^3^ leukocytes, 110,000/mm^3^ erythrocytes, LDH of 1635 IU/L, and amylase of 39 IU/L. Fluid culture was negative. He underwent thoracocentesis and drainage of 2.5 liters of blood tinged fluid that showed LDH 1635 IU/L, Amylase 39 IU/L, RBC 110000/mm^3^, and WBC 6800/mm^3^. Cytology revealed lymphocytic effusion with atypical cells; flow cytometry was inconclusive. Gram stain; bacterial, acid fast, and mycology cultures were all negative.

**Figure 2:**
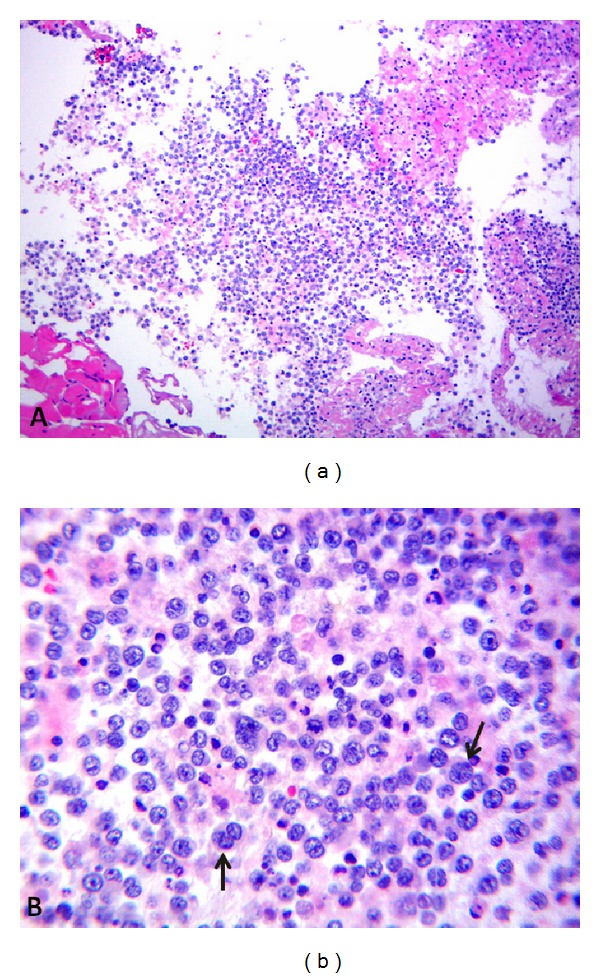
Pathology with H and E stain. Diffuse large B-cell lymphoma (DLBCL), pleural cavity (H&E, (a) 100x, (b) 400x). Aggregates of large atypical lymphocytes with irregular nuclei having uneven chromatin and small to large nucleoli are evident in a necrotic background. Some cells show multilobulated nuclei (arrows). Mitosis and apoptotic bodies are conspicuous.

Three weeks later he presented to the ER with shortness of breath, further weight loss, and worsening malaise. Chest X-ray showed reaccumulation of large left pleural effusion and small right pleural effusion. An unchanged pericardial effusion was again noted. Due to the recurrence of pleural effusion and uncertainty of diagnosis, VATS was done with the intention to drain pleural fluid and for lymph node, pleural, and lung biopsy. After drainage of 2.5 liters of bloody fluid, talc pleurodesis was performed. Lymph node and lung biopsies were negative. Pleural fluid cytology showed atypical large cells with irregular nuclei, vesicular chromatin, and scant to moderate cytoplasm. See Figure 2; immunohistochemical stains were positive for CD-20, CD-10, BCL-2, and kappa restriction and showed a high proliferation index. There was no significant expression of BCL-1 and BCL-6. MIB-1 was expressed in the majority of the cells. The pleural adhesions showed clusters of large necrotic tumour cells and were positive for CD-20. Pathology report was in favour of diffuse large B-cell lymphoma (DLBCL).

Further imaging studies including CT abdomen/pelvis and whole body PET scan did not reveal any lymphadenopathy, organomegaly, or extra cavitary malignancy. Bone marrow biopsy was normal. Final diagnosis was HHV8-unrelated HIV negative primary effusion lymphoma PEL-like lymphoma. Since the patient was CD 20 positive, he was offered chemotherapy with Rituximab, but he declined treatment.

He was monitored very closely for any recurrence of symptoms or relapse of malignancy. A PET scan was repeated a year afterwards and did not show any evidence of disease recurrence. The disease remains in complete remission till date.

## 2. Discussion

Primary effusion lymphoma (PEL) is the least common of the AIDS-related lymphomas, accounting for less than 1 to 4 percent of AIDS-related NHL [1, 2]. It is a B-cell neoplastic process triggered by infection of the tumor clone by human herpesvirus type-8/Kaposi's sarcoma-associated herpesvirus (HHV-8/KSHV). First described in 1996 in HIV infected individuals who were coinfected with Kaposi's sarcoma-associated herpesvirus (KSHV) or HHV-8 virus, it was included as a separate entity in WHO classification of tumors of hematopoietic and lymphoid tissue in the year 2001. PEL is characterized by liquid growth in fluid-filled body spaces, most commonly occurring in HIV patients [3, 4]. During its entire clinical course, the lymphoma tends to remain localized to the serous body cavities with no formation of solid tumor masses. Recently, there have been a few case reports of a solid variant of PEL as well. Historically, PEL was seen in AIDS patients but recently cases have been reported in other immunosuppressive conditions like solid organ transplants [5, 6] and Hepatitis C infected individuals. Therefore, the epidemiology of PEL points towards a close link with the underlying host immunodeficiency. Unlike HIV and Hepatitis C, HHV-8 has a universal association with PEL. This led to its recognition as an independent lymphoma category by the World Health Organization classification system of hematologic neoplasms in 2001.

The precise B-cell subset from which these cells are derived and the biological mechanisms responsible for its unusual growth pattern (limited to body cavities) are uncertain. It has been suggested that the cells represent a preterminal stage of B-cell differentiation [7]. However, others suggest that the development of PEL is not restricted to one stage of B-cell differentiation and may represent transformation of B-cells at different stages of ontogeny [8]. Recently, Notch1, a member of a transmembrane signal transduction family, was found to be strongly expressed in PEL cell lines as well as in a majority of PEL tumors, raising the possibility that Notch1 may be a downstream effector in HHV-8-mediated lymphomagenesis [9].

A search of the English literature was done through PubMed and Google Scholar using the words “PEL lymphoma,” “body cavity lymphomas,” and “HHV-8-unrelated PEL-like lymphomas.” The search was focused on articles, reviews, and case reports published between January 1990 and September 2013. Till date, around 50 cases of HHV-8-unrelated PEL-like lymphoma have been reported upon review of the literature. HIV status of seven patients was not described. All other cases were HIV negative. 10 patients were EBV positive and 7 were Hepatitis C positive. All of the Hepatitis C positive patients had peritoneal involvement that manifested as lymphomatous ascites. Twenty four patients (including ours) were negative for all three of the HIV, EBV, and Hepatitis C serology. In this review, we are presenting the clinical characteristics, therapy, and outcome of this distinct group in a tabular way (Table 1).

There have been few reports of PEL-like process in patients without HIV or HHV-8 infection. This particular clinical entity has now been labeled as HHV-unrelated PEL-like lymphoma [10, 11]. It also presents as lymphomatous effusion in peritoneal, pleural, and pericardial cavities. Here, we reported a case of PEL-like lymphoma, which is HIV and HHV-8 unrelated. The etiology of PEL-like lymphoma unrelated to HHV-8 is far less clear although it may involve infection by Hepatitis C, EBV, liver cirrhosis, and iatrogenic immunodeficiency. HCV infection, in particular, has been suggested as a possible pathogen, given that it was found in approximately 30% to 40% patients. HCV is believed to induce persistent antigenic stimulation that results in B-cell clonal expansion. Most HCV-associated diseases demonstrated peritoneal involvement and HCV-RNA has also been found in ascitic fluid [12]. EBV was also found in some patients with HHV-8-unrelated PEL-like lymphoma but its presence is not necessary for its development. A review of the literature suggests that rate of association with HCV and EBV was 42% and 19.4%, respectively, and the most involved sites were the peritoneum and pleura; pericardial involvement was least common. However, in the majority of patients with HHV-8-unrelated PEL-like lymphoma, as in our patient, no known pathogens such as HIV, EBV, HCV, or iatrogenic immunodeficiency were identified. The only common finding in cases of PEL-like lymphoma that we came across in the published literature is that most of the patients were elderly, with a median age older than 60 years, in contrast to a median age of 44 years in PEL [13]. As it is well known that immune function decreases in geriatric populations, advanced age may be the primary reason for immunodeficiency in HHV-8-unrelated PEL-like lymphoma. It has also been postulated that multistep genomic abnormalities like c-myc amplification might be involved in the development of HIV-negative, HHV8-unrelated PEL-like lymphoma [14].

## 3. Conclusions

PEL-like lymphoma can occur in people without well-defined immunodeficiency states. If detected early, it is potentially curable. Although immune dysregulation seems to play a causal role in most cases, yet absence of immunosuppression and HCV or EBV infections in our patient emphasizes that pathogenesis of PEL or HHV8-unrelated PEL-like lymphoma largely remains unexplored. Reporting of further cases might help better understand the underlying disease mechanisms and may help devise a standard treatment approach.

## Figures and Tables

**Table 1:** HHV-8 negative, HIV, EBV, and Hep C negative PEL-like lymphomas reported.

Reference	Age/sex	Site	Immunophenotyping	Therapy	Outcome
Terasaki et al. [15]	99/F	**Pleural, pericardium**	CD19, CD20, CD5, CD25, IgM, IgD	Drainage	Alive at 16 months
Wang et al. [16]	79/M	Pleural	CD45, CD20, CD79a, bcl-2, bcl-6, MUM1	**Pleurodesis**	Alive at 55 months
Terasaki et al. [15]	85/M	**Pleural, pericardium**	CD20	None	Alive at 11 months
Inoue et al. [17]	67/F	**Pericardium**	CD20, CD79a	CHOP, MEPP, and DEVIC	Expired in 16 months
Kagoya et al. [18]	74/M	**Pericardium**	CD20	RCHOP	Expired in 7 months
Takahashi et al. [19]	73/M	Pleural, **pericardium**, and peritoneum	CD20	CHOP	Alive at 12 months
Terasaki et al. [20]	68/M	Pleural	CD20, CD79a	RCHOP	Alive at 22 months
Fujisawa et al. [21]	69/M	Pleural, **pericardium**	CD19, CD20, CD5, bcl2, Cyclin D1	THP-COP	Expired in 5 months
Youngster et al. [22]	88/M	Pleural	CD20, CD30, CD79a, CD45	RCHOP	Alive at 11 months
Hermine et al. [11]	52/F	Pleural, **pericardium**	CD19, CD20, CD22, CD45, HLA-DR	Not mentioned	Not mentioned
Ohshima et al. [14]	75/M	Pleural	CD19, CD20, HLA-DR	CHOP	Expired in 15 months
Ohshima et al.* [14]	76/M	Pleural	CD19, CD20, CD10, HLA-DR	None	Alive at 6 months
Ohshima et al.* [14]	32/F	Peritoneum	CD10, CD19, CD20, HLA-DR	CHOP and PBSCT	Alive at 13 months
Ohshima et al.* [14]	81/M	Pleural	CD19, CD20, CD10, CD5, HLA-DR	None	Alive at 2 months
Shimazaki et al. [23]	90/F	Pleural, **pericardium, and** peritoneum	CD20, CD79a, BCL-2	None	Expired in five months
Inoue et al. [24]	70/F	Pleural, **pericardium**	CD19, CD20, CD22, CD24, CD8, CD10, CD38, HLA DR	CHOP and **sobuzoxane**	Alive at 30 months
Fujiwara et al. [25]	75/F	**Pericardium**	CD20, CD79a	CHOP	Alive at 36 months
Nemr et al.* [26]	92/F	Pleural	CD20, CD45, BCL-2	None	Expired in 2 months
Nakamura et al. [27]	51/M	Scrotum	CD45, CD19, CD20, CD79a	Carboplatin, etoposide, mitoxantrone, prednisone, and RT	Alive at 8 months
Saini et al. [28]	87/F	Pleural	CD19, CD45, CD20, CD79a	**Pleurodesis**	Alive at 21 months
Saini et al. [28]	82/F	Pleural	CD20, bcl6, MUM1, PAX 5	Pleural drainage	Expired in 13 months
Matsumoto et al. [29]	90/M	Pleural	CD19, CD20, CD30	R + THP-COP	Alive at 38 months
Matsumoto et al. [29]	87/F	Pleural	CD20, CD30	**Rituximab**	Alive at 32 months
***Our patient***	***76/M***	***Pleural/pericardium***	***CD-20, CD-10, BCL-2, MIB 1***	***None***	***Alive at 14 months***

*Status of Hepatitis C is not described.

Currently, there is no standard chemotherapeutic regimen for its treatment. When employed, chemotherapy has largely been based on CHOP regimen.
